# Editorial: Novel Insights Into Obesity-Related Diseases

**DOI:** 10.3389/fphys.2022.952682

**Published:** 2022-06-23

**Authors:** Xiang Zhang, Yanmin Wang, Sanyuan Hu

**Affiliations:** ^1^ Department of General Surgery, Qilu Hospital of Shandong University, Jinan, China; ^2^ California Medical Innovations Institute, San Diego, CA, United States; ^3^ First Affiliated Hospital of Shandong First Medical University, Jinan, China; ^4^ Shandong University, Jinan, China

**Keywords:** obesity, diabetes, cardiomyopathy, bariatric surgery, bile acids

Obesity is a chronic disease with an excessive amount of or ectopically distributed body fat, which leads to unfavorable cosmetic concern as well as increased risks of type 2 diabetes, cardiovascular diseases, nonalcoholic fatty liver disease (NAFLD) and so forth, which could be named as obesity-related diseases. Both obesity and the related diseases have reached epidemic proportions globally, being a major health, economic and social problem worldwide. It has been estimated that global obesity rates have almost tripled since 1975, and now over 671 million adult individuals are living with obesity (Collaborators et al., 2017; NCD Risk Factor Collaboration, 2017; The Lancet Diabetes and Endocrinology, 2021). Due to large population base, China has the largest number of obese worldwide, with approximately 46% of adults and 15% of children being affected (Wang et al., 2017). Therefore, a comprehensive understanding of the pathogenesis, along with the attempt to prevent or treat obesity and comorbidities is of great importance. In this Research Topic named “*Novel Insights into obesity-related diseases*”, we aimed to gather the latest knowledge from basic research to clinical trials relating to pathogenesis and treatment of obesity and related disorders, in order to shed light on novel strategies for obesity management.

## Bariatric Surgery

Currently, bariatric surgery still stands for the most effective interventions for long-term weight loss and alleviation of obesity-related diseases. Sleeve gastrectomy (SG) and Roux-en-Y gastric bypass (RYGB) are the most commonly performed bariatric surgeries worldwide, accounting for 45.9% and 39.6%, respectively (Angrisani et al., 2017; Pucci and Batterham, 2019). Bariatric surgery is usually recommended for people with a body mass index (BMI) > 35 kg/m^2^ or 30–35 kg/m^2^ with uncontrolled conditions (Zimmet et al., 2011). For Asian people with type 2 diabetes, the criterion should be reduced by 2.5 kg/m^2^ (Dixon et al., 2011). In this Research Topic, the study by Luo et al. focused on Chinese diabetic patients with BMI of 27.5–32.5 kg/m^2^, and reported a good insulin resistance remission at 6 months after laparoscopic SG and RYGB; Compared to SG, RYGB achieved a better diabetes remission (56.23% vs. 29.41%), which is consistent with the prevailing viewpoint that RYGB is more efficient in improving metabolic disorders (Padwal et al., 2011; Puzziferri et al., 2014) while SG has the advantage of low risk in the perioperative period and less long-term complications (Frezza et al., 2009; Dick et al., 2010). It seems to be the first report regarding diabetes control in Chinese patients undergoing SG and RYGB with relatively low BMI. Although bariatric surgery has been listed in a variety of obesity management guidelines, some people are not willing to undertake the surgery. In the US, only 1–2% of the eligible candidates undergo bariatric surgery for obesity each year (Gasoyan et al., 2019). Therefore, some gastric restrictive bariatric devices have been proposed (Vargas et al., 2018) as less invasive alternatives. Wang et al. gave the first detailed review on this topic by introducing efficacy and mechanisms of gastric band, gastric sleeve implant, intragastric balloons and so forth. This review also pointed out that the purely “restrictive” devices may take effect beyond just mechanical restriction, which may be taken into consideration for future device design.

Despite sustained clinical therapeutic effects of bariatric surgery, the underlying mechanisms have not been fully elucidated. Diabetic cardiomyopathy, defined as a distinct disease entity that occurs in diabetic patients independent of primary cardiovascular disease (Jia et al., 2018), can be partially attenuated by SG (Leung et al., 2016). Two studies from the First Affiliated Hospital of Shandong First Medical University in China focused on the mechanisms underlying and found that both AMPK and MAPK signaling pathways were involved in the diabetic cardiomyopathy amelioration after SG based on a rodent model. These findings provide possible approaches to combat diabetic cardiomyopathy in patients. Bile acids are thought to be important mediators for metabolic benefits after bariatric surgery, as they were universally increased in the serum postoperatively (except purely restrictive procedures) (Wang et al., 2019). In the field of bile acid research, the technique for bile acid subtype detection is a necessity as different bile acid subtypes have distinct effects on metabolism. Zhao et al. reviewed most of the popular techniques for bile acid subtype detection, which helped researchers choose the most suitable technique based on research models, diseases as well as economic development and geographical factors. Apart from bile acids, long noncoding RNAs (lncRNAs) are also involved in metabolic improvements following bariatric surgery. Liang et al. previously reported duodenal and jejunal lncRNA alterations after duodenal-jejunal bypass (DJB) (Liang et al., 2017; Liang et al., 2018). In this Research Topic, Liang et al. have extended their work to the ileum, and found that dysregulated ileal lncRNAs were associated with lipid and amino acid metabolism and might contribute to postoperative energy homeostasis reestablishment. Instead of focusing on one single mechanism, the series of studies by Liang et al. used transcriptome analysis, which is a fundamental and powerful tool, to understand the microscopic functional alteration of certain organs after bariatric surgery. This methodology highlights the importance of omics technologies and represents future research direction in the modern era.

## Novel Therapies

Compared to surgery, less invasive interventions are more acceptable. Electroacupuncture is a combination of traditional Chinese acupuncture and modern electrical treatment. The study by Sheng et al. reported that electroacupuncture plus diet control improved the community structure of intestinal flora and generated a therapeutic effect for perimenopausal females with abdominal obesity. The results were interesting and were promising for potential self-treatment at home in the future. Maternal cigarette smoke exposure is a severe issue and may cause preterm birth, low birth weight, and catch-up growth in childhood (Collaco et al., 2017). The study by Zeng et al. provided a potential strategy for reducing metabolic disorders in offspring by adding l-leucine supplement based on rodent models. This represents a novel discovery and worth further clinical verification.

## Pathophysiology of Obesity Related Disorders

Obesity also causes alterations in cardiac metabolism, which make ATP production less efficient, producing cardiac dysfunction (Alpert et al., 2018; Elagizi et al., 2018; Nakamura and Sadoshima, 2020). Adaptations to obesity-related metabolic disturbances induce changes in cardiac energy substrate preference. Recent studies have suggested lactate may represent an important fuel for the myocardium during exercise or myocadiac stress (Murashige et al., 2020; Cluntun et al., 2021). Dong et al. summarized the role of lactate in cardiac metabolism and its relevance to the progression and management of heart diseases, including acute myocardial ischemia and heart failure, and in diabetic state. The new perspective has prompted the research for understanding the role of lactate in cardiac metabolism under both normal physiological and pathological conditions. The effect of NAFLD on the whole system has been well reviewed before. The review by Lu et al., for the first time, concentrated on the metabolic changes within the hepatocytes and gave a detailed description of the pathogenesis and outcomes of the disease. As the ultimate cure for NAFLD, liver transplantation is widely performed. The study by Lai et al. reported an altered microbial composition in liver transplantation patients by using 16S rRNA sequencing of fecal samples. This study emphasized the importance of “gut-liver” axis in maintaining metabolic integrity and suggested that manipulation of gut microbiota may help postoperative recovery.

Obesity and related diseases have been an important health problem in the world. Fortunately, researchers never give up and keep exploring in this field. In this Research Topic, most of the studies concentrate on bariatric surgery including SG, RYGB, DJB, and device implant, aiming to investigate the mechanisms underlying and potential therapeutic targets. Electroacupuncture and l-leucine supplementation, have been reported as new therapies in perimenopausal patients with abdominal obesity and maternal cigarette smoke exposure induced metabolic disorder, respectively. Pathophysiology in heart diseases, NAFLD, and after liver transplantation were also investigated; Lactate and intestinal microbial composition are potential targets for manipulation. In summary, articles in this Research Topic have broadened our horizons and added novel insights into obesity-related diseases.
